# Nitrate transporters in leaves and their potential roles in foliar uptake of nitrogen dioxide^†^

**DOI:** 10.3389/fpls.2014.00360

**Published:** 2014-07-30

**Authors:** Yanbo Hu, Victoria Fernández, Ling Ma

**Affiliations:** ^1^College of Life Science, Northeast Forestry UniversityHarbin, China; ^2^Forest Genetics and Ecophysiology Research Group, School of Forest Engineering, Technical University of MadridMadrid, Spain; ^3^School of Forestry, Northeast Forestry UniversityHarbin, China

**Keywords:** chloride channel gene, nitrate reductase, nitrate transporter, nitrogen dioxide, signal transmission

## Abstract

While plant roots are specialized organs for the uptake and transport of water and nutrients, the absorption of gaseous or liquid mineral elements by aerial plant parts has been recognized since more than one century. Nitrogen (N) is an essential macronutrient which generally absorbed either as nitrate (NO^−^_3_) or ammonium (NH^+^_4_) by plant roots. Gaseous nitrogen pollutants like N dioxide (NO_2_) can also be absorbed by plant surfaces and assimilated via the NO^−^_3_ assimilation pathway. The subsequent NO^−^_3_ flux may induce or repress the expression of various NO^−^_3_-responsive genes encoding for instance, the transmembrane transporters, NO^−^_3_/NO^−^_2_ (nitrite) reductase, or assimilatory enzymes involved in N metabolism. Based on the existing information, the aim of this review was to theoretically analyze the potential link between foliar NO_2_ absorption and N transport and metabolism. For such purpose, an overview of the state of knowledge on the NO^−^_3_ transporter genes identified in leaves or shoots of various species and their roles for NO^−^_3_ transport across the tonoplast and plasma membrane, in addition to the process of phloem loading is briefly provided. It is assumed that a NO_2_-induced accumulation of NO^−^_3_/NO^−^_2_ may alter the expression of such genes, hence linking transmembrane NO^−^_3_ transporters and foliar uptake of NO_2_. It is likely that *NRT1/NRT2* gene expression and species-dependent apoplastic buffer capacity may be also related to the species-specific foliar NO_2_ uptake process. It is concluded that further work focusing on the expression of *NRT1* (*NRT1.1*, *NRT1.7*, *NRT1.11*, *and NRT1.12*), *NRT2* (*NRT2.1*, *NRT2.4*, and *NRT2.5*) and chloride channel family genes (*CLCa* and *CLCd*) may help us elucidate the physiological and metabolic response of plants fumigated with NO_2_.

## Introduction

Nitrate (NO^−^_3_) is the most common form of nitrogen used by plants for growth and development (Bertoni, 2012). Despite the major role of plant roots is absorbing and transporting water and mineral elements, there is abundant evidence showing that nutrients can also be taken up by aerial plant parts (e.g., leaves, fruits and stems) (Eichert and Fernández, 2012; Fernández and Brown, 2013). Foliar applied NO^−^_3_ may be absorbed and assimilated efficiently as shown in several studies carried out with different plant species (e.g., Stiegler et al., 2011; Uscola et al., 2014). Gaseous air pollutants like nitrogen dioxide (NO_2_) can also be deposited into plant leaves and be taken up mainly through stomata (Eichert and Fernández, 2012). NO_2_ molecules may dissolve in the aqueous phase of the apoplastic space being consequently transformed into nitrate (NO^−^_3_) and/or nitrite (NO^−^_2_) by chemical reactions. Thereafter, NO_2_-derived NO^−^_3_ may be transported across the plasma membrane by NO^−^_3_ transporters and reach the cytoplasm for further incorporation into cellular N-compounds and/or storage in vacuoles (Hawkesford et al., 2012). The NO^−^_3_ stored in the vacuoles may be exported to compensate for the consumption of NO^−^_3_ in the metabolic pool (De Angeli et al., 2006), which suggests that vacuolar NO^−^_3_ may largely serve as N buffer for transport processes (Hawkesford et al., 2012). NO^−^_3_/proton antiporters, which may be encoded by chloride channel family (CLC) genes, are responsible for NO^−^_3_ influx into plant vacuoles (Geelen et al., 2000). NO^−^_3_ transmembrane transporters may be expected to play a role after the uptake of exogenous NO_2_ or NO^−^_3_ by the foliage. Several NO^−^_3_ transporters identified in leaves have been demonstrated to be closely correlated with e.g., stomatal opening (Guo et al., 2003), NO^−^_3_ reductase activity (Loqué et al., 2003), accumulation and remobilization of NO^−^_3_ (De Angeli et al., 2006; Fan et al., 2009; Lv et al., 2009). Thereby, such physiological processes may significantly influence and also be affected by the foliar uptake of NO_2_ or NO^−^_3_.

**Figure 1:**
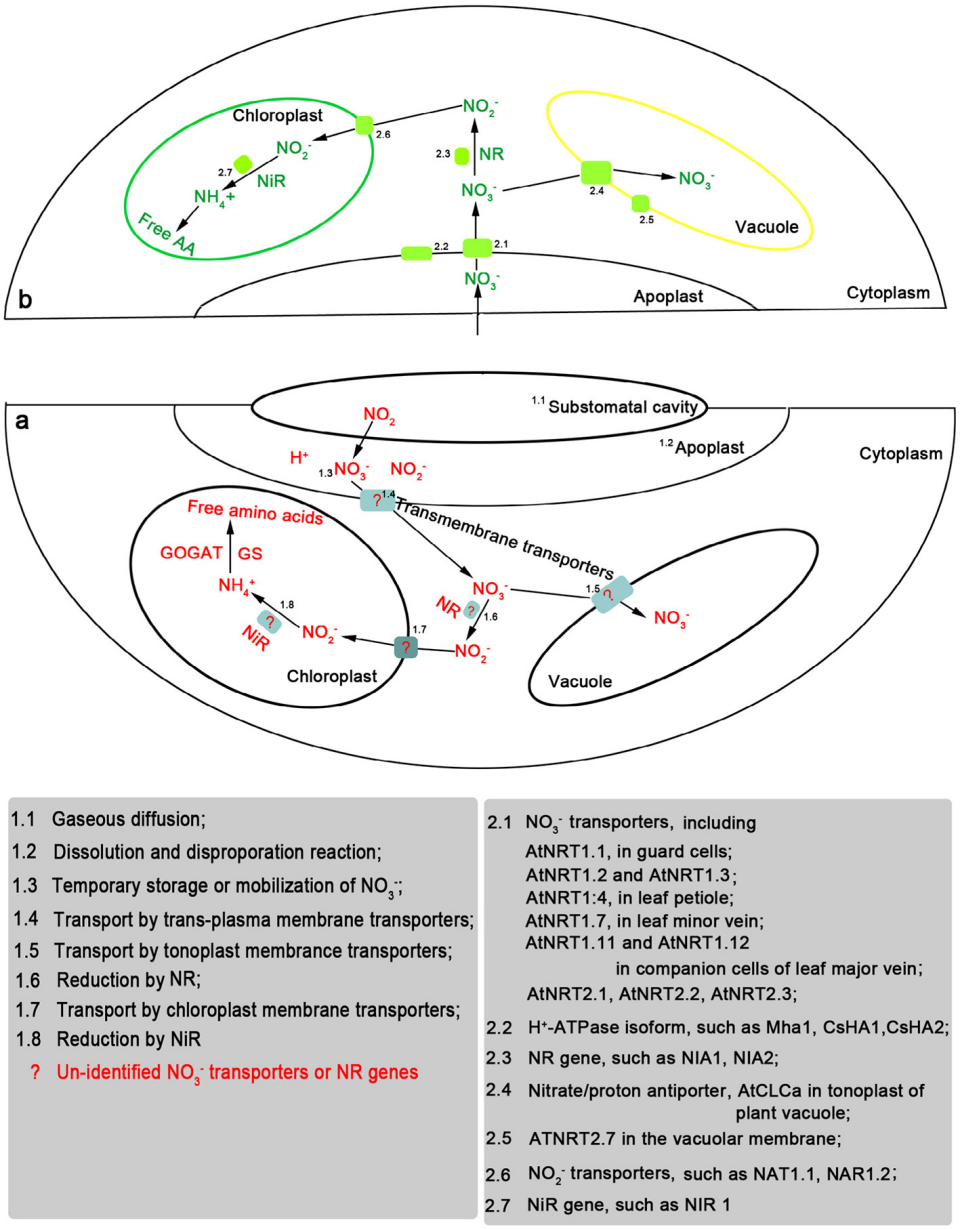
**Nitrate (NO^−^_3_) transporters and NR/NiR genes potentially involved after foliar uptake and assimilation of exogenous nitrogen dioxide (NO_2_) (a)/ NO^−^_3_ (b)**.

Recently some NO^−^_3_ transporter genes were detected in leaves including several members of plant *NRT1* family genes (e.g., *AtNRT1.1*, *AtNRT1.4, AtNRT1.7*, *AtNRT1.11*, and *AtNRT1.12*,.), *NRT2* family genes (e.g., *AtNRT2.1, AtNRT2.3*, *AtNRT2.4*, *AtNRT2.5*, *AtNRT2.6*, *AtNRT2.7, NpNRT2.1* and *ZmNrt2.1*.), and CLC family genes such as *AtClCa* and *AtClCd* (Orsel et al., 2002; Guo et al., 2003; Chopin et al., 2007; Fan et al., 2009; Hsu and Tsay, 2013) (Figure 1). These genes show various expression levels in leaves and play diverse roles in regulating NO^−^_3_ metabolism (Hawkesford et al., 2012). For example, *AtNRT1.1*, a dual-affinity NO^−^_3_-inducible transporter, showed a strong expression in guard cells and supports the stomatal function in the presence of NO^−^_3_ (Guo et al., 2003). *McNRT1*, *LeNRT1.1*, and *NpNRT1.1* were detected in the leaves of *M. crystallinum*, tomato and *Nicotiana plumbaginifolia*, respectively. *McNRT1*, sharing 60% homology with the *AtNRT1.1*, was expressed in the mesophyll cells and cells adjacent to metaxylem vessels in the leaves (Popova et al., 2003). The gene plays potential roles in NO^−^_3_ uptake in the mesophyll cells, distribution and partitioning of NO^−^_3_ within the leaves. Moreover, *AtNRT1.4* and *AtNRT1.7* are of pure low-affinity transporters. *AtNRT1.4* was expressed primarily in the leaf petiole (Chiu et al., 2004). *NRT1.7* mRNA was detected in the distal lamina of older leaves, but not in the roots (Fan et al., 2009). The two transporter genes participate in the process of leaf NO^−^_3_ storage and remobilization. For the members of the *NRT2* family, the amounts of *AtNRT2.4* transcripts were predominant in leaves of the adult plants, followed by *AtNRT2.5*; the expression of *AtNRT2.1*, *AtNRT2.6*, and *AtNRT2.7* were at low levels (Orsel et al., 2002). *AtNRT2.1*, *AtNRT2.3*, *AtNRT2.4*, and *AtNRT2.5* are NO^−^_3_-responsive genes, whereas *AtNRT2.6* and *AtNRT2.7* appear to be constitutive genes (Loqué et al., 2003). Particularly, *AtNRT2.7* showed a strong leaf- and seed-specific expression pattern (Orsel et al., 2002; Chopin et al., 2007), while *AtNRT2.3* was specifically expressed in leaves at a reproductive stage.

Previous studies on foliar uptake of NO_2_ mainly focused on the deposition pathways, metabolic processes associated with NO_2_-derived NO^−^_3_ (Hu, 2011; Hu et al., 2014), and downstream products of NO_2_-N assimilation (Nussbaum et al., 1993; Weber et al., 1995). The current state of knowledge on the potential plant responses to NO_2_ exposure is summarized in Table 1. However, the relationship between NO^−^_3_ transmembrane transporters and foliar NO_2_ uptake has received only limited scientific attention so far. Foliar uptake of NO_2_ seems to be species-specific and concentration-dependent (Hu and Sun, 2010). Expression of the genes encoding leaf NO^−^_3_ transporters also appears to be species-specific (Ono et al., 2000; Orsel et al., 2002). The contribution of various expression patterns of transporter genes to species-specific NO_2_ uptake is currently unknown. Given the N transport mechanisms described above, a potential relationship between foliar NO_2_ uptake (substomatal build-up of NO_2_ and the subsequent reduction, storage, and remobilization of NO^−^_3_) and NO^−^_3_-responsive genes encoding the transmembrane transporters and NO^−^_3_/NO^−^_2_ reductases may be hypothesized. For validating such hypothesis, future work focusing on the relationship between organ-specific expression of NRT1/NRT2 genes and species-specific NO_2_ uptake should be carried out.

**Table 1:** **Physiological and metabolic responses of plant organs to nitrogen dioxide (NO_2_) exposure**.

**Plant organ**	**Action site**	**Physiological function of exogenous NO_2_ on plants**		**References**
		**Low NO_2_ concentration (e.g., 40–60 nl.l^−1^)**	**High NO_2_ concentration (e.g., 1–4 μl.l^−1^)**	
Leaf	Stomata	Stimulation on stomatal aperture and stomatal conductance^[1.3]^;	Stomatal closure and declined stomatal conductance^[1.1]^	^[1.1]^Qiao and Murray, 1998;
		Reduced stomatal density^[1.2]^		^[1.2]^Siegwolf et al., 2001;
				^[1.3]^Takagi and Gyokusen, 2004
	Apoplast	Increase in the malondialdehyde (MDA) level and superoxide dismutase (SOD) at 0.5 μ L.L^−1^ NO^[2.1]^_2_	Acidity of apoplast^[1.1]^;	^[2.1]^Ma et al., 2007;
			Induced expression of germin-like proteins (RmGLP2) ^[2.2]^;	^[2.2]^Kondo et al., 2008;
			Decline in MDA content and SOD activity^[2.3]^;	^[2.3]^Chen et al., 2010
			Decline in ASA^[2.1]^	
	Chloroplast	Increase in NR, NiR^[3.4]^, photosynthetic rate^[3.5]^, and chlorophyll content, etc.	Decline in chlorophyll content, ratio of Fv/Fm^[2.3]^, and apparent photosynthesis^[3.1]^;	^[3.1]^Srivastava et al., 1974;
				^[3.2]^Yoneyama et al., 1979;
			Accumulation of NO^−^_3_ and NO^−[3.2]^_2_ as well as increase in NR and NiR^[3.2]^; Inhibition of NR^[3.3]^	^[3.3]^Hisamatsu et al., 1988;
				^[3.4]^Weber et al., 1995
				^[3.5]^Schmutz et al., 1995
	Mitochondria/ Peroxisome		Inhibition of dark respiration and apparent photorespiration^[3.1, 4.2]^;	^[4.1]^Dolzmann and Ullrich, 1966;
			Protrusions from both plastids and mitochondria of *Phaseolus vulgaris* exposed to NO_2_ (10 ml.l^−1^)^[4.1]^	^[4.2]^Carlson, 1983
	In developing or maturing leaves	Increased leaf area^[1.2]^;	NO_2_-N incorporation into free amino acids such as glutamine, glutamic acid, γ-amino butyric acid and aspartic acid^[5.1]^;	^[5.1]^Yoneyama and Sasakawa, 1979;
		NO_2_-N incorporation into free amino acids such as Glu, Asp and Gln^[3.4; 5.3]^;		^[5.2]^Schiffgens-Gruber and Lutz, 1992
		Stimulation on cell proliferation and enlargement as well as up-regulation of the related genes, such as ARGOS, GRF5, and KLU^[5.4]^	NO_2_ led to swollen thylakoids and a reduction in the number of grana stacks^[5.2]^	^[5.3]^Nussbaum et al., 1993;
				^[5.4]^Takahashi et al., 2014
Stems	Xylem	Enlarged width of xylem in the main stem of Poplar trees^[3.5]^	stem growth significantly decreased by NO_2_ at 1.0 μ l.l^−1^ ^[6.1]^	^[6.1]^Eastham and Ormrod, 1986
	Phloem	NO_2_-N incorporation into free amino acids of bark of Norway spruce^[5.3]^	NO_2_-N incorporation into free amino acids such as serine, asparagine and glutamine^[6.2]^	^[6.2]^Wellburn, 1990
Roots		NO_2_-N incorporation into free amino acids in Norway spruce roots^[5.3]^	Decrease in root/shoot ratio, dry matter production, concentration of soluble sugars in roots, root respiration of kidney bean plants ^[7.1]^	^[7.1]^Ito et al., 1985;
			Decrease in root nitrate uptake in sunflower plants^[7.2]^ and soybean plants^[1.1]^, increase in the ammonium concentration in roots of soybean plants at 1.1 μ l.l^−1^ NO^[1.1]^_2_	^[7.2]^Okano et al., 1985
Flowers		Acceleration of flowering time and increase in flower number^[5.4; 8.1]^		^[8.1]^Takahashi et al., 2011;
Fruits		Increased fruit yield^[8.1]^ or grain yield (the number and weight of grain) and protein stored (at NO_2_ of 170 nl.1^−1^)^[9.1]^		^[9.1]^Murray et al., 1994

## Substomatal build-up of NO_2_ may disturb apoplastic pH and NO^−^_3_ transporters

The apoplast is defined as the area within the plant tissues which is beyond the cell plasma membrane, and includes the cell wall, middle lamella, xylem and gas and water filled intercellular spaces (Sattelmacher, 2001). The leaf apoplastic space plays a role in ion exchange and as a diffusion barrier (Sattelmacher, 2001). Estimates of the volume of leaf water in the apoplast vary from 10 to 35% of total leaf water (Speer and Kaiser, 1991; Wardlaw, 2005). Dissolution of NO_2_ in the apoplast may produce H^+^ and NO^−^_3_/NO^−^_2_. Foliar NO_2_ uptake is calculated to yield at most 0.22 mol excess H^+^ per mol N (Raven, 1988). Therefore, a build-up of NO_2_ in the leaf substomatal cavities may lead to apoplastic pH disturbances. Among other factors, the resulting apoplastic pH changes will depend on NO_2_ concentration, root N supply and plant N status. The supply of NO^−^_3_ via the root system significantly increased the leaf apoplastic pH of *Phaseolus vulgaris* and *Helianthus annuus*, whereas the depletion of NO^−^_3_ in nutrient solution led to lower leaf apoplastic pH values in *Zea mays* (Mühling and Lauchli, 2001). NH_4_NO_3_ nutrition did not change the leaf apoplastic pH in sunflower (Kosegarten et al., 1999). Moreover, foliar NH^+^_4_ fertilization may either lead to apoplastic alkalinization (Felle and Hanstein, 2002) or acidification (Mühling and Lauchli, 2001). When supplying NH_4_C1 (1 mM) via the root to soybean plants, low concentrations of NO_2_ (0.2–0.25 μL· L^−1^) significantly increased the leaf apoplastic pH (Qiao and Murray, 1997), whereas under a higher root NO^−^_3_ dose (5 mM), high concentrations of NO_2_ (1.1 μ l· l^−1^) increased the acidity of the leaves (Qiao and Murray, 1998). Apoplastic pH is an important factor affecting plasmalemma proton pumps (Hoffmann et al., 1992; Sattelmacher, 2001). Apoplastic alkalinization or acidification may induce plasma membrane depolarization or hyperpolarization (Hedrich et al., 2001). This may further modulate the deactivation or activation of membrane-bound proton-transporting enzymes, and the corresponding ion channel regulation for co-transport of anions (Savchenko et al., 2000). Wippel et al. (2010) found that the fluctuation of apoplastic pH had a regulatory effect on plant sucrose transporters. Based on the above information, it can be reckoned that the apoplastic pH changes caused by NO_2_ may repress or induce NO^−^_3_-responsive genes encoding the transmembrane transporters.

Some NO^−^_3_ transporter genes (such as *AtNRT1.1* and *ZmNrt2.1*) in leaves are NO^−^_3_-inducible, while others such as *AtNRT2.5* are NO^−^_3_-repressible (Okamoto et al., 2003). Low-affinity transporter systems (*NRT1* family) may significantly contribute to NO^−^_3_ uptake at external NO^−^_3_ concentrations above 250 μ M. However, high-affinity transporters (*NRT2* family) including the constitutive (cHATS Km = 6–20 mM) and inducible HATS (Km = 20–100 mM), are active at low external concentrations of 0–0.5 mM (Crawford and Glass, 1998; Quaggiotti et al., 2003; Hawkesford et al., 2012). When analyzing the *AtNRT1.7* NO^−^_3_ transporter gene *in Arabidopsis*, Fan et al. (2009) applied 50 mM K^15^NO_3_ to carry out measurements on distal parts of the rosette leaf. The ^15^N-NO^−^_3_ tracing assay showed that the percentage of total ^15^N in the leaves ranged from 0 to 10% for wild-type plants, and between 5 and 15% for the *nrt1.7* mutants. The percentage of NO^−^_3_–^15^N was in the range of the NO_2_-derived reduced N of wild herbaceous plants (from 0.98 to 10.1%) and woody plants (0.15–12.7%) for the 217 taxa fumigated with 4.0 ± 0.1 μmol· mol^−1^ NO_2_ (Morikawa et al., 1998). Accordingly, the content of NO_2_-derived reduced N ranged from 0.25 to 5.72 mg N· g^−1^ dry weight for wild herbaceous plants, and 0.04–6.57 mg N· g^−1^ dry weight for woody plants. This comparison suggests that the amounts of NO_2_-derived NO^−^_3_ in leaves are in the range of the NO^−^_3_ concentrations which may induce the two types of transporter systems (i.e., high and low affinity).

From the reasoning provided above, it can be reckoned that substomatal build-up of NO_2_ may lead to concentration-dependent changes of apoplastic pH and NO^−^_3_ concentration. Such pH fluctuations may influence NO^−^_3_ transmembrane transport by the induction or repression of the transporters and transporter gene expression, and may provide some sort of feedback regulation on the uptake of NO_2_ by the foliage. For example, apoplastic mesophyll signals have been recently found to induce rapid stomatal responses in *Commelina communis* (Fujita et al., 2013). In response to NO_2_ fumigation, multiple physiological and metabolic responses may occur which could either ultimately favor or inhibit the process of symplastic N uptake (Table 1). The multi-responses of NO^−^_3_ transporters to the substomatal build-up of NO_2_ may partially contribute to species-specific NO_2_ uptake, but future studies with different plant species shall be carried out for clarifying this complex issue.

## Nitrate transporters are possibly involved in the reduction and accumulation of NO_2_-derived NO^−^_3_

In leaf cytoplasm, NO_2_-derived NO^−^_3_ has at least two fates: (i) assimilation into amino acids, and (ii) accumulation in vacuole (Hawkesford et al., 2012). The metabolic pathway will depend on the external NO^−^_3_ concentration and leaf N demand (Stulen et al., 1998). NO_2_-derived NO^−^_3_ will be assimilated mainly through the NO^−^_3_ assimilation pathway (Morikawa et al., 1998). NO^−^_3_ reductase (NR) is considered as a key rate-limiting enzyme of NO_2_-N assimilation (Hawkesford et al., 2012). A linear correlation was found between NR activity, NO_2_ concentration and amounts of N incorporated into amino acids (Sparks et al., 2001). However, high levels of NO_2_ fumigation resulted into a loss of NR activity or a rapid inactivation of the leaf NR (Takeuchi et al., 1985; Hisamatsu et al., 1988), and NO^−^_3_ accumulation (Ma et al., 2007). This down-regulation of the NR may be ascribed to at least one of the following phenomena: (i) a high NO_2_ concentration will inhibit the activities of glutamine synthetase and glutamate synthase, which leads to NH^+^_4_ accumulation and subsequently brings about a loss of NR activity (Orebamjo and Stewart, 1975; Padidam et al., 1991), and (ii) a high NO_2_-induced stomatal closure may lead to a rapid NR inactivation due to a low CO_2_ availability. High NO_2_ rapidly induced stomatal closure (Qiao and Murray, 1998). Stomatal closure may trigger a chain reaction wherein the lower CO_2_ availability will lead to the subsequent leaf NR activity decrease (Kaiser and Forster, 1989). *NIA1* and *NIA2* genes encode the two isoforms of the NR apoprotein (Wilkinson and Crawford, 1993). Recent reports show a close relationship between the expression of *NIA1*/*NIA2* genes and *NRT1/NRT2* genes. Addition of external NO^−^_3_ strongly induced the expression of genes encoding the NR (*AtNIA1* and *AtNIA2*) and the transmembrane transporters (*AtNRT1.1*, *AtNRT2.1*, *NpNRT2.1*; Fraisier et al., 2001; Jonassen et al., 2009). In contrast, high levels of external NO^−^_3_ caused a down-regulation of *AtNRT1.1* and *AtNIA1* through a pathway of NO^−^_2_-induced repression (Loqué et al., 2003). This down-regulation of the *AtNRT1.1* gene is associated with a decrease in the NO^−^_3_ influx. Earlier studies showed that high NO_2_ concentration fumigation under light or dark conditions resulted in leaf NO^−^_2_ accumulation (Yoneyama and Sasakawa, 1979; Yu et al., 1988). Thus, we may assume that a high NO_2_-caused NO^−^_2_ accumulation may lead to a negative feedback regulation on leaf NO_2_ uptake through the down-regulation of the *NRT1.1* gene and the subsequent repression of the NO^−^_3_ influx. Moreover, studies on NR mutants showed that *AtNRT1.1*, *AtNRT1.7*, *NpNRT2.1*, and *AtNIA1* are up-regulated in NR-deficient mutants (*NIA2*- and/or *MoCo* biosynthesis-deficient mutants) (Lejay et al., 1999; Vidmar et al., 2000; Fan et al., 2009). Under NR-repressible or -deficient conditions, this up-regulation of the transporter genes may be beneficial to an exportation of excess NO^−^_3_ in the leaf (Fan et al., 2009). Moreover, Jonassen et al. (2009) demonstrated that the bZIP transcription factors *HY5* and *HYH* regulate positively *NIA2* gene and negatively *NRT1.1* gene. However, *HY5* and *HYH* appear to be mediated by light but not by external NO^−^_3_.

Excess NO^−^_3_ may be accumulated in leaf vacuole (Hawkesford et al., 2012). The H^+^/NO^−^_3_ antiport across tonoplast is responsible for NO^−^_3_ influx and H^+^/NO^−^_3_ symport for NO^−^_3_ efflux (Figure 1). The flux direction will depend on the requirements and conditions of the cell (Schumaker and Sze, 1987). Three members of the chloride channel family (CLC) genes *AtClCa* (De Angeli et al., 2006), *AtClCc* (Harada et al., 2004) and *AtClCd* (Lv et al., 2009) have been identified in the leaf tonoplast. De Angeli et al. (2009) demonstrated that adenosine triphosphate (ATP) induces a negative regulation on *AtCLCa* activity. NO_2_ fumigation significantly increased ATP amounts of *Lolium perenne* and *Phleum pratense*, the amounts increasing with raising NO_2_ concentrations (Wellburn et al., 1981). This may be due to the formation of free radicals in response to NO_2_ fumigation, which may damage photosynthetic membranes and hence alter the proton gradients to which ATP formation is linked (Wellburn et al., 1981). Yoneyama and Sasakawa (1979) found that 8 ppm NO_2_ fumigation under dark conditions resulted in NO^−^_2_ accumulation in spinach leaves. High doses of NO^−^_2_ resulted in the peroxidation of lipid constituents of chloroplastic membrane (Ezzine and Ghorbel, 2006). Chen et al. (2010) found that leaf uptake of NO_2_ reduced the rate of photosynthesis and increased the malondialdehyde (MDA) concentration, may be due to a competition for nicotinamide adenine dinucleotide phosphate (NADPH) between the processes of NO^−^_2_ reduction vs. carbon assimilation, and the generation of reactive oxygen species (ROS) (Sabaratnam and Gupat, 1988; Shimazaki et al., 1992).

## Transmembrane transporters in leaves mediating NO^−^_3_ signaling

NO_2_ fumigation may significantly disrupt plant morphology and physiology by, for instance, changing the shoot to root ratio, stomatal, and gas exchange dynamics, or modifying root N uptake (Qiao and Murray, 1997, 1998; Table 1). The exposure of plants to NO_2_ increased the total content of soluble free amino acids in leaves and shoots (Nussbaum et al., 1993). Most of the amino acids may be used locally for the synthesis of e.g., Chlorophyll and Rubisco during rapid vegetative growth, or be ultimately designated for e.g., filling pods (Imsande and Touraine, 1994). NO^−^_3_ assimilation products (protein/nucleic acids and amino acids/amides) can also be transferred into roots under soil N deficit (Wellburn, 1990). Under a low NO^−^_3_ supply, gaseous NO_2_ may change the amino acid ratio of the xylem. For example, the amount of serine, asparagine and glutamine were high in the xylem of plants exposed to atmospheric NO_2_, whereas arginine, cysteine, valine and lysine were high in the control plants (Wellburn, 1990; Table 1). Moreover, NO_2_ treatment increased phloem transport of organic N and inhibited the rate of xylem N translocation.

NO_2_-N metabolism and the mobilization of metabolic products will trigger various signaling pathways that regulate the physiological and metabolic processes. The dissolution of NO_2_ and subsequent reduction can result in root NO^−^_3_ uptake changes. The xylem is part of leaf apoplast (Felle and Hanstein, 2002). Thus, NO_2_-caused apoplastic pH changes may serve as a signal to modify the uptake of NO^−^_3_ via the root system. This NO^−^_3_ signaling pathway has been explained by Qiao and Murray (1998). Moreover, NO^−^_3_ reduction can produce malate (Touraine et al., 1988); the organic acid needs to be membrane transported to be loaded into the phloem. A transport of malate from leaves to roots can serve as another signal to control root uptake of NO^−^_3_ (Touraine et al., 1992). This signaling pathway has been reported by Imsande and Touraine (1994). Tonoplast transport of malate plays an important role in physiological regulation in NO^−^_3_ nutrition (Hawkesford et al., 2012). At the cellular level, NO^−^_3_ accumulation led to increased expression of genes encoding organic acid synthesis (*PPC*, cytosolic PK, CS, *ICDH-1*) and accumulation of malate and a-oxoglutarat. In contrast, leaf malate supply can inhibit *NIA* expression, affecting both the *NIA* transcript level and the activity (Müller et al., 2001).

NO^−^_3_ itself may serve as a signaling molecule (Scheible et al., 1997). NO^−^_3_ addition to the growing media can induce or repress the expression of various genes encoding e.g., NO^−^_3_ transporters, NO^−^_3_/NO^−^_2_ reductase, ferredoxin reductase, and the enzymes in the pentose phosphate pathway, or iron or sulfate transport and metabolism (Wang et al., 2003; Marschner, 2012). The expression of NO^−^_3_-responsive genes [such as NADH-specific and NAD(P)H-bispecific NR genes] is dependent upon NO^−^_3_ flux but not on the NO^−^_3_ amount stored in the tissue (Gojon et al., 1991). Excess external NO^−^_3_ may be stored in several vacuoles and recirculated after storage (Hawkesford et al., 2012). NO^−^_3_ remobilization may occur among different organs, for instance, from older leaves to younger leaves during the vegetative stage or from leaves to seeds during the reproductive stage (Schiltz et al., 2005; Hawkesford et al., 2012). N remobilization is rate-limited by the transport of NO^−^_3_ across tonoplast of vacuole, plasma membrane of mesophyll cell, plasma membrane of companion cell and sieve element, and phloem loading (Fan et al., 2009). CLC genes (*AtClCa*, *AtClCc*, and *AtClCd*) are required for the transport of NO^−^_3_ across tonoplast in vacuole. Disruption of one of these genes will influence the flux of NO^−^_3_ in vacuoles (De Angeli et al., 2006). *AtNRT2.4* showed a strong induction in a low NO^−^_3_ provision. Orsel et al. (2004) suggested that *AtNRT2.4* and *AtNRT2.5* participate in the transport of NO^−^_3_ from stored pools (vacuoles) to cytoplasm. Moreover, *AtNRT2.7* was also involved in this type of NO^−^_3_ flux; this gene could play roles in leaf balance between the amount of NO^−^_3_ used for assimilation and that re-absorbed for further transport (Orsel et al., 2002). Four NRT1 family genes (*AtNRT1.4, AtNRT1.7*, *AtNRT1.11*, and *AtNRT1.12*) participate in the phloem- and/or xylem-loading of NO^−^_3_ (Figure 1). *AtNRT1.4* was expressed predominantly in the leaf petiole and involved in petiole NO^−^_3_ accumulation (Chiu et al., 2004). The mutation of the *AtNRT1:4* resulted in significant changes of NO^−^_3_ content in leaf petiole and the lamina. Furthermore, the deficiency of *AtNRT1.4* can alter leaf development. *NRT1.7* was expressed in the phloem of the leaf minor vein and mediated the remobilization of excess NO^−^_3_ from older leaves to younger leaves (Fan et al., 2009). Compared with the wild-type plants, the *nrt1.7* null mutants accumulated a higher amount of NO^−^_3_ in the older leaves and decreased the NO^−^_3_ content of phloem exudates from older leaves. The newly identified NO^−^_3_ transporters (NRT1.11 and *NRT1.12*) were expressed in the companion cells of the major vein (Hsu and Tsay, 2013). They play roles in xylem-to-phloem transfer for redistributing NO^−^_3_ into developing leaves. Moreover, several NRT2 genes may also be involved in the N remobilization. For example, *ZmNrt2.1* plays a potential role in NO^−^_3_ loading from the xylem or in its compartmentation (Quaggiotti et al., 2003).

## Conclusion

The substomatal build-up of NO_2_ and subsequent NO^−^_3_ metabolism may lead to apoplastic alkalinization or acidification and to NO^−^_3_/NO^−^_2_ concentration fluctuations in the leaf apoplast and symplast (e.g., cytoplasm and vacuole), which depend on NO_2_ concentration and root N supply. These changes will cause complex responses of NO^−^_3_-responsive genes encoding NO^−^_3_ transporters and NO^−^_3_/NO^−^_2_ reductase. For example, addition of external NO^−^_3_ produced a strong induction on NR genes (such as *AtNIA1* and *AtNIA2*) and the transporter genes (such as *AtNRT1.1*, *AtNRT2.1*, and *NpNRT2.1*). However, excess NO^−^_2_ significant inhibited the expression of *AtNRT1.1* and *AtNIA1*, and disturbed CLC family genes by regulating the generation of ATP. This down-regulation of the *NRT1.1* gene is associated with a decrease in NO^−^_3_ influx. Moreover, *AtNRT2.4*, *AtNRT2.5*, and *AtNRT2.7* may participate in the transfer of NO^−^_3_ from stored pools (vacuoles) to cytoplasm. *AtNRT1.4*, *AtNRT1.7*, *AtNRT1.11*, and *AtNRT1.12* are involved in the phloem- and/or xylem-loading of NO^−^_3_. Thus, these genes are suggested to play rate-limiting roles in foliar uptake of NO_2_. Further work is proposed to investigate the relationship between organ specificity of NRT1/NRT2 gene expression and species-specific NO_2_ uptake.

In practical terms, a high rate of low concentration NO_2_ absorption by the foliage may be positive for preserving an adequate plant N status. However, a high NO_2_ concentration may alter leaf apoplast chemistry, leading to the accumulation of NO^−^_3_ and NO^−^_2_, and providing signals which may negatively affect plant N nutrition. These factors are however closely linked with leaf NO^−^_3_ transporters and may also interact with the foliar uptake processes (e.g., by promoting stomatal closure). Thereby, a low NO_2_ concentration may act as a positive regulation signal (Takahashi et al., 2014) by stimulating the leaf NO^−^_3_ transporters, and enhancing NO^−^_3_ transport and distribution. In contrast, a high NO_2_ concentration in relation to a high rate of foliar NO_2_ absorption, may repress the expression of NO^−^_3_ transporters and enzymes, which may protect the cells or organelles from NO_2_ damage.

## Author contributions

Yanbo Hu organized and wrote the original manuscript; Yanbo Hu and Victoria Fernandez discussed and revised the revising manuscript and approved the final version; Ling Ma collected the information for Table 1, and went through the manuscript.

### Conflict of interest statement

The authors declare that the research was conducted in the absence of any commercial or financial relationships that could be construed as a potential conflict of interest.

## References

[B1] Bertoni G. (2012). A nitrate transporter for both roots and shoots. Plant Cell 24, 1 10.1105/tpc.112.240110 22227892 PMC3289557

[B1a] Carlson R. W. (1983). Interaction between SO_2_ and NO_2_ and their effects on photosynthetic properties of soybean *Glycine max*. Environ. Pollut. Ecol. Biol. 32, 11–38 10.1016/0143-1471(83)90071-5

[B2] Chen Z. M. Chen Y. X. Du G. J. Wu X. L. Li F. (2010). Effects of 60-day NO_2_ fumigation on growth, oxidative stress and antioxidative response in *Cinnamomum camphora* seedlings. J. Zhejiang Univ. Sci. B 11, 190–199 10.1631/jzus.B0910350 20205305 PMC2833403

[B3] Chiu C. C. Lin C. S. Hsia A. P. Su R. C. Lin H. L. Tsay Y. F. (2004). Mutation of a nitrate transporter, AtNRT1:4, results in a reduced petiole nitrate content and altered leaf development. Plant Cell Physiol. 45, 1139–1148 10.1093/pcp/pch143 15509836

[B4] Chopin F. Orsel M. Dorbe M. F. Chardon F. Truong H. N. Miller A. J. (2007). The Arabidopsis AtNRT2.7 nitrate transporter controls nitrate content in seeds. Plant Cell 19, 1590–1602 10.1105/tpc.107.050542 17540716 PMC1913726

[B5] Crawford N. M. Glass A. D. M. (1998). Molecular and physiological aspects of nitrate uptake in plants. Trends Plant Sci. 10, 389–395 10.1016/S1360-1385(98)01311-9

[B6] De Angeli A. Monachello D. Ephritikhine G. Frachisse J. M. Thomine S. Gambale F. (2006). The nitrate/proton antiporter AtCLCa mediates nitrate accumulation in plant vacuoles. Nature 442, 939–942 10.1038/nature05013 16878138

[B7] De Angeli A. Moran O. Wege S. Filleur S. Ephritikhine G. Thomine S. (2009). ATP binding to the C terminus of the *Arabidopsis thaliana* nitrate/proton antiporter, AtCLCa, regulates nitrate transport into plant vacuoles. J. Biol. Chem. 284, 26526–26532 10.1074/jbc.M109.005132 19636075 PMC2785341

[B8] Dolzmann P. Ullrich H. (1966). Einige Beobachtungen iiber Beziehungen zwischen Chloroplasten und Mitchondrien im Palisadenparenchym von *Phaseolus vulgaris*. Zeitschrift fiir Pflanzenphysiologie 55, 165–180

[B9] Eastham A. M. Ormrod D. P. (1986). Visible injury and growth responses of young cuttings of *Populus Canadensis* and *P. nigra* to nitrogen dioxide and sulphur dioxide. Can J. For. Res. 16, 1289–1292 10.1139/x86-228

[B10] Eichert T. Fernández V. (2012). Uptake and release of elements by leaves and other aerial plant parts, in Marschner's Mineral Nutrition of Higher Plants, ed Marschner P. (Oxford: Academic Press), 71–84

[B11] Ezzine M. Ghorbel M. H. (2006). Physiological and biochemical responses resulting from nitrite accumulation in tomato. (*Lycopersicon esculentum* Mill. cv. Ibiza F1). J. Plant Physiol. 163, 1032–1039 10.1016/j.jplph.2005.07.013 16971215

[B12] Fan S. C. Lin C. S. Hsu P. K. Lin S. H. Tsay Y. F. (2009). The *Arabidopsis* nitrate transporter NRT1.7, expressed in phloem, is responsible for source-to-sink remobilization of nitrate. Plant Cell 21, 2750–2761 10.1105/tpc.109.067603 19734434 PMC2768937

[B13] Felle H. H. Hanstein S. (2002). The apoplastic pH of the substomatal cavity of *Vicia faba* leaves and its regulation responding to different stress factors. J. Exp. Bot. 53, 73–82 10.1093/jexbot/53.366.73 11741043

[B14] Fernández V. Brown P. H. (2013). From plant surface to plant metabolism: the uncertain fate of foliar-applied nutrients. Front. Plant Sci. 4:289 10.3389/fpls.2013.00289 23914198 PMC3728483

[B15] Fraisier V. Dorbe M. F. Daniel-Vedele F. (2001). Identification and expression analyses of two genes encoding putative low-affinity nitrate transporters from *Nicotiana plumbaginifolia*. Plant Mol. Biol. 45, 181–190 10.1023/A:1006426616760 11289509

[B16] Fujita T. Noguchi K. Terashima I. (2013). Apoplastic mesophyll signals induce rapid stomatal responses to CO_2_ in Commelinacommunis. New Phytol. 199, 395–406 10.1111/nph.12261 23560389

[B17] Geelen D. Lurin C. Bouchez D. Frachisse J. M. Lelièvre F. Courtial B. (2000). Disruption of putative anion channel gene AtCLC-a in *Arabidopsis* suggests a role in the regulation of nitrate content. Plant J. 21, 259–267 10.1046/j.1365-313x.2000.00680.x 10758477

[B18] Gojon A. Wakrim R. Passama L. Robin P. (1991). Regulation of NO^–^_3_ assimilation by anion availability in excised soybean leaves. Plant Physiol. 96, 398–405 10.1104/pp.96.2.398 16668199 PMC1080783

[B19] Guo F. Q. Young J. Crawford N. M. (2003). The nitrate transporter AtNRT1.1 (CHL1) functions in stomatal opening and contributes to drought susceptibility in *Arabidopsis*. Plant Cell 15, 107–117 10.1105/tpc.006312 12509525 PMC143464

[B20] Harada H. Kuromori T. Hirayama T. Shinozaki K. Leigh R. A. (2004). Quantitative trait loci analysis of nitrate storage in Arabidopsis leading to an investigation of the contribution of the anion channel gene, AtCLC-c, to variation in nitrate levels. J. Exp. Bot. 55, 2005–2014 10.1093/jxb/erh224 15310822

[B21] Hawkesford M. Horst W. Kichey T. Lambers H. Schjoerring J. Skrumsager I. (2012). Functions of Macronutrients, in Marschner's Mineral Nutrition of Higher Plants, 3rd Edn., eds Marschner P. (London: Elsevier), 135–178

[B22] Hedrich R. Neimainis S. Savchenko G. Felle H. H. Kaiser W. M. Heber U. (2001). Changes in apoplastic pH and membrane potential in leaves in relation to stomatal responses to CO_2_, malate, abscisic acid or interruption of water supply. Planta 213, 594–601 10.1007/s004250100524 11556792

[B23] Hisamatsu S. Nihira J. Takeuchi Y. C. Satoh S. Kondo N. (1988). NO_2_ suppression of light-induced nitrate reductase in squash cotyledons. Plant Cell Physiol. 29, 395–401

[B24] Hoffmann B. Plänker R. Mengel K. (1992). Measurements of pH in the apoplast of sunflower leaves by means of fluorescence. Physiol. Plant. 84, 146–153 10.1111/j.1399-3054.1992.tb08777.x10594095

[B25] Hsu P. K. Tsay Y. F. (2013). Two phloem nitrate transporters, NRT1.11 and NRT1.12, are important for redistributing xylem-borne nitrate to enhance plant growth. Plant Physiol. 163, 844–856 10.1104/pp.113.226563 24006285 PMC3793062

[B26] Hu Y. B. (2011). NO_2_-drived NO^−^_3_ metabolism in leaves. Insci. J. 1, 90–101 10.5640/insc.010290

[B27] Hu Y. B. Bellaloui N. Sun G. Y. Tigabu M. Wang J. H. (2014). Exogenous sodium sulfide improves morphological and physiological responses of a hybrid Populus species to nitrogen dioxide. J. Plant Physiol. 171, 868–875 10.1016/j.jplph.2013.10.018 24635903

[B28] Hu Y. B. Sun G. Y. (2010). Leaf nitrogen dioxide uptake coupling apoplastic chemistry, carbon/sulfur assimilation, and plant nitrogen status. Plant Cell Rep. 29, 1069–1077 10.1007/s00299-010-0898-5 20628880

[B29] Imsande J. Touraine B. (1994). N demand and the regulation of nitrate uptake. Plant Physiol. 105, 3–7 12232181 10.1104/pp.105.1.3 PMC159322

[B30] Ito O. Okano K. Kuroiwa M. Totsuka T. (1985). Effects of NO_2_ and O_3_ alone or in combination on kidney bean plants (*Phaseolus vulgaris* L.): partitioning of assimilates and root activities. J. Exp. Bot. 36, 652–662 10.1093/jxb/36.4.652

[B31] Jonassen E. M. Sevin D. C. Lillo C. (2009). The bZIP transcription factors HY5 and HYH are positive regulators of the main nitrate reductase gene in *Arabidopsis* leaves, NIA2, but negative regulators of the nitrate uptake gene NRT1.1. J. Plant Physiol. 166, 2071–2076 10.1016/j.jplph.2009.05.010 19540016

[B32] Kaiser W. M. Forster J. (1989). Low CO_2_ prevents nitrate reduction in leaves. Plant Physiol. 91, 970–974 10.1104/pp.91.3.970 16667163 PMC1062103

[B32a] Kondo K. Yamada K. Nakagawa A. Takahashi M. Morikawa H. Sakamoto A. (2008). Molecular characterization of atmospheric NO_2_-responsive germin-like proteins in azalea leaves. Biochem. Biophys. Res. Commun. 377, 857–861 10.1016/j.bbrc.2008.10.060 18950603

[B33] Kosegarten H. U. Hoffmann B. Mengel K. (1999). Apoplastic pH and Fe^3+^ reduction in intact sunflower leaves. Plant Physiol. 121, 1069–1079 10.1104/pp.121.4.1069 10594095 PMC59475

[B34] Lejay L. Tillard P. Lepetit M. Olive F. D. Filleur S. Daniel-Vedele F. (1999). Molecular and functional regulation of two NO^−^_3_ uptake systems by N- and C-status of Arabidopsis plants. Plant J. 18, 509–519 10.1046/j.1365-313X.1999.00480.x 10417701

[B35] Loqué D. Tillard P. Gojon A. Lepetit M. (2003). Gene expression of the NO^−^_3_ transporter NRT1.1 and the nitrate reductase NIA1 is repressed in Arabidopsis roots by NO^−^_2_, the product of NO^−^_3_ reduction. Plant Physiol. 132, 958–967 10.1104/pp.102.018523 12805624 PMC167034

[B36] Lv Q. D. Tang R. J. Liu H. Gao X. S. Li Y. Z. Zheng H. Q. (2009). Cloning and molecular analyses of the *Arabidopsis thaliana* chloride channel gene family. Plant Sci. 176, 650–661 10.1016/j.plantsci.2009.02.006

[B37] Ma C. Y. Xu X. Hao L. Cao J. (2007). Nitrogen dioxide-induced responses in *Brassica campestris* seedlings: the role of hydrogen peroxide in the modulation of antioxidative level and induced resistance. Agric. Sci. China 6, 1193–1200 10.1016/S1671-2927(07)60163-1

[B38] Marschner P. (2012). Mineral Nutrition of Higher Plants. San Diego, CA: Academic Press

[B39] Morikawa H. Higaki A. Nohno M. Takahashi M. Kamada M. Nakata M. (1998). More than a 600-fold variation in nitrogen dioxide assimilation among 217 plant taxa. Plant Cell Environ. 21, 180–190 10.1046/j.1365-3040.1998.00255.x

[B40] Mühling K. H. Lauchli A. (2001). Influence of chemical form and concentration of nitrogen on apoplastic pH of leaves. J. Plant Nutr. 25, 399–411 10.1081/PLN-100104968

[B41] Müller C. Scheible W. R. Stitt M. Krapp A. (2001). Influence of malate and 2-oxoglutarate on the NIA transcript level and nitrate reductase activity in tobacco leaves. Plant Cell Environ. 24, 191–203 10.1111/j.1365-3040.2001.00664.x

[B42] Murray F. Wilson S. Samaraweera S. (1994). NO_2_ increases wheat grain yield even in the presence of SO_2_. Agric. Ecosyst. Environ. 48, 115–123 10.1016/0167-8809(94)90082-5

[B43] Nussbaum S. Ballmoos P. V. Gfeller H. Schlunegger U. P. Fuhrer J. Rhodes D. (1993). Incorporation of atmospheric ^15^NO_2_-nitrogen into free amino acids by Norway spruce *Picea abies* (L.) Karst. Oecologia 94, 408–414 10.1007/BF00317117 28313679

[B44] Okamoto M. Vidmar J. J. Glass A. D. (2003). Regulation of NRT1 and NRT2 gene families of Arabidopsis thaliana: responses to nitrate provision. Plant Cell Physiol. 44, 304–317 10.1093/pcp/pcg036 12668777

[B45] Okano K. Totsuka T. Fukuzawa T. Tazakl T. (1985). Growth responses of plants to various concentrations of nitrogen dioxide. Environ. Pollut. 38, 361–373 10.1016/0143-1471(85)90107-2

[B46] Ono F. Frommer W. B. von Wire'n N. (2000). Coordinated diurnal regulation of low- and high-affinity nitrate transporters in tomato. Plant Biol. 2, 17–23 10.1055/s-2000-297

[B47] Orebamjo T. O. Stewart G. R. (1975). Ammonium inactivation of nitrate reductase in *Lemna minor* L. Planta 122, 37–44 10.1007/BF00385402 24435919

[B48] Orsel M. Eulenburg K. Krapp A. Daniel-Vedele F. (2004). Disruption of the nitrate transporter genes AtNRT2.1 and AtNRT2.2 restricts growth at low external nitrate concentration. Planta 219, 714–721 10.1007/s00425-004-1266-x 15107992

[B49] Orsel M. Krapp A. Daniel-Vedele F. (2002). Analysis of the NRT2 nitrate transporter family in *Arabidopsis*: structure and gene expression. Plant Physiol. 129, 886–896 10.1104/pp.005280 12068127 PMC161709

[B50] Padidam M. Venkateswarlu K. Johri M. M. (1991). Ammonium represses NADPH nitrate reductase in the moss *Funaria hygrometrica*. Plant Sci. 75, 185–194 10.1016/0168-9452(91)90233-X

[B51] Popova O. V. Dietz K. J. Golldack D. (2003). Salt-dependent expression of a nitrate transporter and two amino acid transporter genes in *Mesembryanthemum crystallinum*. Plant Mol. Biol. 52, 569–578 10.1023/A:1024802101057 12956527

[B52] Qiao Z. Murray F. (1997). Effects of atmospheric nitrogen dioxide on uptake and assimilation of ammonium in soybean plants. J. Plant Nutr. 20, 1183–1190 10.1080/01904169709365326

[B53] Qiao Z. Murray F. (1998). The effects of NO_2_ on the uptake and assimilation of nitrate by soybean plants. Environ. Exp. Bot. 10, 33–40 10.1016/S0098-8472(97)00023-324272470

[B54] Quaggiotti S. Ruperti B. Borsa P. Destro T. Malagoli M. (2003). Expression of a putative high-affinity NO^−^_3_ transporter and of an H^+^-ATPase in relation to whole plant nitrate transport physiology in two maize genotypes differently responsive to low nitrogen availability. J. Exp. Bot. 54, 1023–1031 10.1093/jxb/erg106 12598572

[B55] Raven J. A. (1988). Acquisition of nitrogen by the shoots of land plants: its occurrence and implication for acid-base regulation. New Phytol. 109, 1–20 10.1111/j.1469-8137.1988.tb00212.x

[B56] Sabaratnam S. Gupat G. (1988). Effects of nitrogen dioxide on biochemical and physiological characteristics of soybean. Environ. Pollut. 55, 149–158 10.1016/0269-7491(88)90125-X 15092510

[B57] Sattelmacher B. (2001). The apoplast and its significance for plant mineral nutrition. New Phytol. 149, 167–192 10.1046/j.1469-8137.2001.00034.x 33874640

[B58] Savchenko G. Wiese C. Neimanis S. Hedrich R. Heber U. (2000). pH regulation in apoplastic and cytoplasmic cell compartments of leaves. Planta 211, 246–255 10.1007/s004250000280 10945219

[B59] Scheible W. R. Gonzalez-Fontes A. Lauerer M. Muller-Rober B. Caboche M. Stitt M. (1997). Nitrate acts as a signal to induce organic acid metabolism and repress starch metabolism in tobacco. Plant Cell 9, 783–798 10.1105/tpc.9.5.783 12237366 PMC156956

[B60] Schiffgens-Gruber A. Lutz C. (1992). Ultrastructure of mesophyll cell chloroplasts of spruce needles exposed to O_3_, SO_2_ and NO_2_ alone and in combination. Environ. Exp. Bot. 32, 243–254 10.1016/0098-8472(92)90007-O

[B61] Schiltz S. Munier-Jolain N. Jeudy C. Burstin J. Salon C. (2005). Dynamics of exogenous nitrogen partitioning and nitrogen remobilization from vegetative organs in pea revealed by ^15^N *in vivo* labeling throughout seed filling. Plant Physiol. 137, 1463–1473 10.1104/pp.104.056713 15793068 PMC1088335

[B62] Schmutz P. Tarjan D. Günthardt-Goerg M. S. Matyssek R. Bucher J. B. (1995). Nitrogen dioxide- a gaseous fertilizer of Poplar trees. Phyton 35, 219–232

[B63] Schumaker K. S. Sze H. (1987). Decrease of pH gradients in tonoplast vesicles by NO^−^_3_ and Cl^−^: evidence for H^+^-coupled anion transport. Plant Physiol. 83, 490–496 10.1104/pp.83.3.490 16665277 PMC1056392

[B64] Shimazaki K. Yu S. W. Sakaki T. Tanaka K. (1992). Differences between spinach and kidney bean plants in terms of sensitivity to fumigation with NO_2_. Plant Cell Physiol. 33, 267–273

[B65] Siegwolf R. T. W. Matyssek R. Saurer M. Maurer S. Günthardt-Goerg M. S. Schmutz P. (2001). Stable isotope analysis reveals differential effects of soil nitrogen and nitrogen dioxide on the water use efficiency in hybrid poplar leaves. New Phytol. 149, 233–246 10.1046/j.1469-8137.2001.00032.x 33874623

[B66] Sparks J. P. Monson R. K. Sparks K. L. Lerdau M. (2001). Leaf uptake of nitrogen dioxide (NO_2_) in a tropical wet forest: implications for tropospheric chemistry. Oecologia 127, 214–221 10.1007/s004420000594 24577652

[B67] Speer M. Kaiser W. M. (1991). Ion relations of symplastic and apoplastic space in leaves from Spinacia oleracea L. and Pisum sativum L. under salinity. Plant Physiol. 97, 990–997 10.1104/pp.97.3.990 16668541 PMC1081114

[B68] Srivastava H. S. Jolliffe P. A. Runeckles V. C. (1974). Inhibition of gas exchange in bean leaves by NO_2_. Can. J. Bot. 53, 466–474 10.1139/b75-057

[B69] Stiegler J. C. Richardson M. D. Karcher D. E. (2011). Foliar nitrogen uptake following urea application to putting green turfgrass species. Crop Sci. 51, 1253–1260 10.2135/cropsci2010.06.0377

[B70] Stulen I. Perez-Soba M. De Kok L. J. Van Der Eerden L. (1998). Impact of gaseous nitrogen deposition on plant functioning. New Phytol. 139, 61–70 10.1046/j.1469-8137.1998.00179.x

[B71] Takagi M. Gyokusen K. (2004). Light and atmospheric pollution affect photosynthesis of street trees in urban environments. Urban For. Urban Green. 2, 167–171 10.1078/1618-8667-00033

[B72] Takahashi M. Furuhashi T. Ishikawa N. Horiguchi G. Sakamoto A. Tsukaya H. (2014). Nitrogen dioxide regulates organ growth by controlling cell proliferation and enlargement in Arabidopsis. New Phytol. 201, 1304–1315 10.1111/nph.12609 24354517

[B73] Takahashi M. Sakamoto A. Ezura H. Morikawa H. (2011). Prolonged exposure to atmospheric nitrogen dioxide increases fruit yield of tomato plants. Plant Biotechnol. 8, 485–487 10.5511/plantbiotechnology.11.0819a

[B74] Takeuchi Y. C. Nihira J. Kondo N. Tezuka T. (1985). Change in Nitrate-reducing activity in squash seedlings with NO_2_ fumigation. Plant Cell Physiol. 26, 1027–1035

[B75] Touraine B. Grignon N. Grignon C. (1988). Charge balance in NO^–^_3_ fed soybean: estimation of K^+^ and carboxylate recirculation. Plant Physiol. 88, 605–612 10.1104/pp.88.3.605 16666356 PMC1055632

[B76] Touraine B. Muller B. Grignon C. (1992). Effect of phloem-translocated malate on NO^−^_3_ uptake by roots of intact soybean plants. Plant Physiol. 99, 1118–1123 10.1104/pp.99.3.1118 16668978 PMC1080591

[B77] Uscola M. Villar-Salvador P. Oliet J. Warren C. (2014). Foliar absorption and root translocation of nitrogen from different chemical forms in seedlings of two Mediterranean trees. Environ. Exp. Bot. 104, 34–43 10.1016/j.envexpbot.2014.03.004

[B78] Vidmar J. J. Zhuo D. Siddiqi M. Y. Schjoerring J. K. Touraine B. Glass A. D. M. (2000). Regulation of high-affinity nitrate transporter genes and high-affinity nitrate influx by nitrogen pools in roots of barley. Plant Physiol. 123, 307–318 10.1104/pp.123.1.307 10806247 PMC59004

[B79] Wang R. C. Okamoto M. Xing X. Crawford N. M. (2003). Microarray analysis of the nitrate response in Arabidopsis roots and shoots reveals over 1000 rapidly responding genes and new linkages to glucose, trehalose-6-phosphate, iron, and sulfate metabolism. Plant Physiol. 132, 556–567 10.1104/pp.103.021253 12805587 PMC166997

[B80] Wardlaw I. F. (2005). Viewpoint: consideration of apoplastic water in plant organs: a reminder. Funct. Plant Biol. 32, 561–569 10.1071/FP04127 32689156

[B81] Weber P. Nussbaum S. Fuhrer J. Gfeller H. Schlunegger U. P. Brunold C. (1995). Uptake of atmospheric ^15^NO_2_ and its incorporation into free amino acids in wheat (*Triticum aestivum* L.). Physiol. Plant 94, 71–77 10.1111/j.1399-3054.1995.tb00786.x

[B82] Wellburn A. R. (1990). Why are atmospheric oxides of nitrogen usually phytotoxic and not alternative fertilizers? New Phytol. 115, 395–429 10.1111/j.1469-8137.1990.tb00467.x 33874286

[B83] Wellburn A. R. Higginson C. Robinson D. Walmsley C. (1981). Biochemical explanations of more than additive inhibitory effects of low atmospheric levels of sulphur dioxide plus nitrogen dioxide upon plants. New Phytol. 88, 223–237 10.1111/j.1469-8137.1981.tb01719.x

[B84] Wilkinson J. Q. Crawford N. M. (1993). Identification and characterization of a chlorate-resistant mutant of *Arabidopsis* thaliana with mutations in both nitrate reductase structural genes NIA1 and NIA2. Mol. Gen. Genet. 239, 289–297 8510658 10.1007/BF00281630

[B85] Wippel K. Wittek A. Hedrich R. Sauer N. (2010). Inverse pH regulation of plant and fungal sucrose transporters: a mechanism to regulate competition for sucrose at the host/pathogen interface? PLoS ONE 5:e12429 10.1371/journal.pone.0012429 20865151 PMC2928750

[B86] Yoneyama T. Sasakawa H. (1979). Transformation of atmospheric NO_2_ absorbed in spinach leaves. Plant Cell Physiol. 20, 263–266

[B87] Yoneyama T. Sasakawa H. Ishizuka S. Totsuka T. (1979). Absorption of atmospheric NO_2_ by plants and soils. Soil Sci. Plant Nutr. 25, 267–275 10.1080/00380768.1979.10433167

[B88] Yu S. W. Li L. Shimazaki K. I. (1988). Response of spinach and kidney bean plants to nitrogen dioxide. Environ. Pollut. 55, 1–13 10.1016/0269-7491(88)90155-8 15092511

